# Measuring cognitive load: mixed results from a handover simulation for medical students

**DOI:** 10.1007/s40037-015-0240-6

**Published:** 2016-01-12

**Authors:** John Q. Young, David M. Irby, Maria-Louise Barilla-LaBarca, Olle ten Cate, Patricia S. O’Sullivan

**Affiliations:** Department of Psychiatry, Hofstra North Shore-LIJ School of Medicine, Zucker Hillside Hospital, Glen Oaks, NY USA; Department of Medicine, UCSF School of Medicine, San Francisco, USA; Department of Medicine, Hofstra North Shore-LIJ School of Medicine, Hempstead, NY USA; Center for Research & Development of Education, University Medical Center Utrecht, Utrecht, The Netherlands; Research and Development in Medical Education, UCSF School of Medicine, San Francisco, USA

**Keywords:** Measurement, Cognitive Load, Handover, Validity, Cognitive Load Theory

## Abstract

**Introduction:**

The application of cognitive load theory to workplace-based activities such as patient handovers is hindered by the absence of a measure of the different load types. This exploratory study tests a method for measuring cognitive load during handovers.

**Methods:**

The authors developed the Cognitive Load Inventory for Handoffs (CLI4H) with items for intrinsic, extraneous, and germane load. Medical students completed the measure after participating in a simulated handover. Exploratory factor and correlation analyses were performed to collect evidence for validity.

**Results:**

Results yielded a two-factor solution for intrinsic and germane load that explained 50 % of the variance. The extraneous load items performed poorly and were removed from the model. The score for intrinsic load correlated with the Paas Cognitive Load scale (*r* = 0.31, *p* = 0.004) and was lower for students with more prior handover training (*p* = 0.036). Intrinsic load did not, however, correlate with performance. Germane load did not correlate with the Paas Cognitive Load scale but did correlate as expected with performance (*r* = 0.30, *p* = 0.005) and was lower for those students with more prior handover training (*p* = 0.03).

**Conclusions:**

The CLI4H yielded mixed results with some evidence for validity of the score from the intrinsic load items. The extraneous load items performed poorly and the use of only a single item for germane load limits conclusions. The instrument requires further development and testing. Study results and limitations provide guidance to future efforts to measure cognitive load during workplace-based activities, such as handovers.

## Essentials

- Cognitive load theory focuses on how extraneous, intrinsic, and germane load impacts the working memory of a learner.
- Given the absence of validated instruments, this study tests a method for measuring cognitive load during handovers, the Cognitive Load Inventory for Handoffs (CLI4H).
- The CLI4H yielded mixed results. There was some evidence for validity for the intrinsic and germane load items but not for the extraneous load items.
- These results offer encouragement that cognitive load types, with additional development and testing, can be measured during handovers.
- Methodological lessons from the study provide guidance to others conducting research and developing methods in the areas of handoffs and cognitive load theory.

## Introduction

Handovers, or the transfer of clinical information and responsibility from one clinician or team to another, occur frequently in health care. These transitions in care are vulnerable to communication failures that often lead to medical errors and harm to patients [1]. In response to this hazard, considerable attention has focused on interventions to improve patient safety during handovers [2], many of which were adapted from industries such as nuclear power and space aviation in which transition errors have high consequences [3]. These best practices aim to ensure that the necessary information is transmitted via communication protocols that include structured face-to-face and written sign-out, interactive questioning, and distraction-free settings [4].

Interventions that deploy these practices simultaneously (often referred to as a bundle) have yielded significant improvements in educational and clinical outcomes [5]. Medical schools and residency programmes are rapidly implementing handoff curricula that teach these best practices [2]. However, even with these gains, errors continue to occur during patient handovers, often in the form of information loss (e.g., drug allergy, critical comorbidity, relevant history or current treatments) or distortion (e.g., wrong medication dose, wrong surgical site, or incorrect diagnosis). Information loss and distortion increases when the cognitive load of the handover exceeds the working memory capacity of the clinician sender and/or receiver. To further improve patient safety will require a deeper understanding of human cognition in order to identify the challenges trainees face when learning how to give and receive sign-outs and to use this understanding to design an assessment that can help identify novel intervention targets and measure their efficacy.

Human memory consists of three main subsystems: sensory memory, working memory, and long-term memory [6]. Sensory memory perceives and briefly retains visual and auditory information [7]. Sensory information raised to conscious awareness enters the domain of working memory. Working memory retrieves relevant knowledge possessed by the learner and stored in long-term memory as schemata. Working memory then organizes and integrates the new with the already existing information to facilitate efficient storage in the form of new (or modified) schemata [8].

Originally developed by John Sweller in the context of studying how students problem solve [9], cognitive load theory (CLT) focuses on the implications of limited working memory for learning [10]. Unlike sensory and long-term memory, working memory is not infinite—it can only hold a limited number of independent information units at a time (4–7 ± 2) [11] and can actively process (i.e. organize, compare and contrast) no more than two to four elements at any given moment [8]. CLT researchers have distinguished between different types of cognitive load. In 1998, John Sweller argued for three types [12]:

1. Intrinsic—load associated with the task itself (i.e., working memory resources required to process the information essential to the task). Intrinsic load depends on the number of information elements, the interactivity of those elements, and the knowledge of the learner.
2. Extraneous—load not essential to the task but induced by the design of the task (e.g., how the information is presented) or the environment (e.g., background noise).
3. Germane—load imposed by the learner’s deliberate use of cognitive strategies to refine existing schemata and enhance storage in long-term memory.

Recent work by Sweller and others has suggested that germane load may best be understood as a component of intrinsic load rather than a separate type of load [13, 14]. In this view, a two-factor model (intrinsic and extraneous load) is preferred on theoretical grounds and best explains empirical results.

Given working memory limitations and the still developing schemata of trainees, the additive effects of these different types of load can easily exceed the working memory capacity of the trainee, resulting in impaired learning and performance. Regardless of how germane load is conceptualized, CLT uses three strategies to enhance learning: reduce extraneous load, titrate intrinsic load to the developmental stage of the learner, and increase germane load.

Researchers have developed a number of techniques to estimate cognitive load [15, 16], including learner self-rating of effort [16–24], response time to a secondary task (e.g., visual monitoring task) presented during the primary task [16, 18], and psychophysiological measures (e.g. heart rate variability, pupillary response, and electrical skin conductance) [20]. Secondary task performance and physiological measures only capture overall cognitive load, but are not dependent on learner perception and can capture in real time how load may dynamically change over the course of the task. Learner self-rating has been the most commonly used strategy because it is inexpensive and has evidence of validity [25]. Paas developed a single item designed to measure overall cognitive load [22]. This measure has been used extensively, including in a recent study on cognitive load and surgical knot tying [26], but may actually measure intrinsic load rather than overall load [27, 28]. The NASA-TLX measures mental workload with a multi-item scale [21, 29]. It is unclear to what extent mental workload corresponds to cognitive load [13, 27].

The use of instruments that measure only overall load has presented challenges. For example, integrating visual and written information has been shown to reduce overall load and improve learning [30]. Some have assumed that this occurs due to decreased extraneous load [31] while others have argued that the benefits of data integration are also mediated by increased germane load [32]. The absence of measures of specific load types permits competing and sometimes contradictory explanations to exist in parallel. To address this challenge and further develop CLT, researchers have tested instruments that attempt to differentiate cognitive load types [13, 16–20, 24, 28]. To date, these studies are of variable methodological quality, focus mostly on classroom-based learning settings and have shown better results for items intended to capture intrinsic and extraneous load and only mixed results for germane load items [13].

The most promising efforts to collect validity evidence for a measure of load types have focused on content-specific learning (e.g., college statistics) in the classroom setting [13, 28]. This measure has recently been adapted for use in two medical education studies, though neither reports validity evidence for use of the measure in this context [33, 34]. In addition, Naismith et al. discuss how their own measure of load types compares with the Paas overall measure and the NASA-TLX [27]. The authors identified the need for the development of validity evidence of measures appropriate for workplace-based clinical procedures, in general, and handovers, in particular. Such measures are necessary to identify the cognitive mechanisms of current handover interventions and to develop new handover strategies that modulate intrinsic, extraneous, and germane loads in the desired directions. The authors developed a novel measure, the Cognitive Load Inventory for Handoffs (CLI4H). This measure was then tested in the context of a handover simulation that medical students completed during a multi-station objective structured clinical skills examination (OSCE). In order to provide evidence in support of the validity of the scores from this measure of cognitive load, the study addressed the following questions:

1. To what extent does the CLI4H yield factors consistent with intrinsic, extraneous and/or germane load?
2. How does the performance of the CLI4H compare with the Paas Cognitive Load scale—a single-item measure of cognitive load with evidence to support validity? Positive correlations would support construct alignment between the two measures.
3. Do the CLI4H scores vary, as predicted by CLT, with measures of amount of training and performance? According to CLT, students with greater prior training should experience lower intrinsic and germane load while students with higher performance should experience lower intrinsic load and higher germane load.

## Methods

### Design

This is a psychometric study of the CLI4H. Data were collected according to the framing of validity as a unitary concept [35] and therefore focused on collecting validity evidence from several sources: content of the items themselves as determined by expert input, internal structure via exploratory factor analysis, and correlation with other variables [35]. We did not collect two important classes of evidence identified by the unitary framework, namely response process and consequential validity.

### Participants

In the final weeks of the academic year, all second-year (*n*=54) and third-year (*n*=33) students at the lead author’s medical school participated in a required six-station OSCE that simulated the clinical story of a patient from presentation to admission. Each student completed the stations in the following order: (1) interview of a standardized patient, (2) oral presentation to an attending, (3) interpretation of related diagnostic tests, (4) documentation of the findings and assessment and plan, (5) verbal sign-out of the patient to a standardized resident, and (6) reflection on the experience. The study focused on the ‘sender’ only in the handover, the fifth station in this process. Most students had prior experience with handovers, because the curriculum initiates clinical experiences from the beginning of the first year and also requires all students to function throughout medical school as a licensed emergency medical technician who gives a handover with every patient. This exercise was performed in a clinical skills lab. Institutional Review Board approval was obtained.

### Cognitive load measure

To develop measures of intrinsic, extraneous, and germane load, we examined prior studies [16–19, 23] with special attention to two recent studies with promising results [13, 28]. The last two studies tested a questionnaire with 3 or 4 items for each subtype of cognitive load. The questionnaires yielded a three-factor solution with similar factor loadings and explained more than 75 % of the total variance. While the items intended to measure germane load functioned as a single factor, they did not correlate with performance, leading the authors to question whether this factor reflected the construct of germane load. We adopted the scale (i.e., 0–10 with anchors such as ‘not at all’ for 0 and ‘completely’ for 10) and the basic structure of these items. However, the items were created for classroom-based instruction on non-clinical topics (e.g., statistics and language). For example, the items related to statistics session were focused on ‘topics’, ‘formulas’, and ‘concepts and definitions’—pedagogical constructs specific to statistics [28]. To adapt the content, the authors built upon recent work that proposes the major drivers of intrinsic, extraneous, and germane load in handovers [36]. Items measuring intrinsic load focused on the patient’s complexity, acuteness which was hypothesized to increase intrinsic load by compressing time for clinical decision-making, volume of clinical information and, finally, the extent to which clinical decisions to be made after the handoff involve multiple, interacting information elements. Extraneous load items addressed the accessibility/fragmentation of the information, distractions, and how well the protocol and the terminology were understood. Initial germane load items included concentration [37] and improvements in understanding [13, 19, 28]. These items were then reviewed by two experts in CLT, three experts in handovers, and the study authors [38]. Three clinicians were asked to review the items and explain their understanding of what each item was asking. The items were modified and, in the end, a nine-item measure was developed with four questions each on intrinsic and extraneous load (Table 1). The validity evidence for measures of germane load has been less consistent and robust. The current controversy around how and whether to measure germane load separately from intrinsic load made developing consensus on this part of the instrument challenging. Expert reviewers could only agree on a single item on the extent to which the activity improved understanding.

**Table 1:** Cognitive Load Inventory for Handoffs (CLI4H)

Type of cognitive load	Items	Anchors for 0–10 Scale	
		0	10
Intrinsic load	Please rate the COMPLEXITY of the patient in this handoff	NOT at all complex	Extremely complex
	Please rate the ACUTENESS of the patient in this handoff	Not at all acute	Highly acute
	Please rate the AMOUNT OF CLINICAL INFORMATION that needed to be communicated during this handoff	Low amount of clinical information	High amount of clinical information
	Please rate the CLINICAL DECISIONS that will have to be made after this handoff	Not at all complex	Highly complex, possibly depending on multiple pieces of interacting information.
Extraneous load	Please rate how well you understood the HANDOFF PROTOCOL that was used	Not at all	Completely
	Please rate the ACCESSIBILITY of all the different information that needed to be communicated	Highly fragmented and difficulty to organize	Readily accessible and easy to organize
	Please rate how much MENTAL EFFORT you invested to UNDERSTAND the TERMINOLOGY used in the handoff	Extremely low	Extremely high
	Please rate how DISTRACTED you were during the handoff	Not at all	Very much
Germane load	Please rate how much this handoff improved YOUR UNDERSTANDING of how to perform a handoff?	Not at all	Very much

### Other variables

In order to compare the performance of the new items with a previously published item, we included the Paas Cognitive Load Scale, a single item designed to measure overall cognitive load with a nine-point scale (ranging from extremely low to extremely high). Previous studies provide evidence of validity for the score from this measure [39], though recent work suggests that the Paas score may correlate more with intrinsic load than overall load [13, 26, 27]. We also included items on year of training, prior handover experience (i.e., estimated number of handovers participated in during training as a medical student) and the student’s self-assessment of how successful the handover in the simulation was on a 10-point scale ranging from ‘not at all’ to ‘very successful’. Because the distribution of the data was bimodal, prior experience was subsequently defined as low (five or less prior handovers) and high (more than five). After the student performed the handover, the standardized resident used a five-item checklist to rate whether the student performed each of the five components of the handoff protocol in which they had been trained: illness severity (stable, unstable, critical), summary statement, active issues, if-then contingency planning, and follow-up activities. The measure utilized a three-point scale 0 for ‘No’, 1 for ‘Partial’, and 2 ‘Yes’.

### Procedures

Two weeks prior to the simulation, students received a 2-h training in the handover protocol described above, which was adapted from two published methods for oral communication during a handover [2, 40]. Students were asked to use that method during the handover station. Upon completion of the simulated handover, the standardized residents rated the quality of the handover while the students progressed to the next and final station at which they completed the survey that included the CLI4H, the Paas measure, prior handover experience, self-assessment of the success of the handover, and a prompt to reflect on how the station helped with handovers. Actors with the clinical skills laboratory were trained for their role as standardized residents, including how to use the performance checklist.

### Analysis

The Kaiser-Meyer-Olkin (KMO) measure of sampling adequacy was calculated [41]. We chose to assess the internal structure of the items with exploratory factor analysis rather than confirmatory factor analysis, because the items had never been tested before, were created for a novel setting, and differed considerably from items in previously published work. Moreover, we had a sample size well below the requirements for confirmatory factor analysis. Varimax rotation with Kaiser normalization, pair-wise deletion was performed to test whether the CLI4H yielded a three-factor pattern. While cognitive load items do tend to inter-correlate, we considered varimax to be appropriate given the theoretical argument that load types are independent of one another. Factors with Eigen values exceeding 1 were considered. Factor loading > 0.5 were used to identify items characteristic of the factor [41]. We created scores for the resulting factors by summing the items that composed the factor. To test whether learning/performance and experience were correlated with the cognitive load factors, bivariate Pearson correlations were computed between the scores created for each cognitive load factor and the students’ self-assessment of the handover’s success, the standardized residents’ rating of performance, and the total number of handovers performed during their training to date. Two-tailed t-tests were performed to compare the factor scores for second- versus third-year students and those with low (less than 5) versus high (five or more) handover experience. Level of significance was set at 0.05 for each test. SPSS (version 22) was used for statistical tests.

## Results

### Descriptive analysis

100 % of second-year (*n* = 54) and third-year (*n* = 33) students participated in the OSCE, including the handover station. Second-year students were 50 % female with a mean age of 26.3 (standard deviation, 3.7) while third-year students were 45 % female with a mean age of 27.2 (standard deviation, 3.2). Of the students, 52 had ‘low’ experience while 34 had ‘high’ experience. Our two measures for experience (year in medical school and number of prior handovers completed) co-varied. Therefore, we eliminated year in medical school from subsequent analyses because we believe that number of prior handovers serves as a better approximation of experience with handovers. Missing data were minimal (one third-year student’s questionnaire and another third-year student’s ratings from the standardized resident); these two students were eliminated from the analyses.

### Validity evidence related to internal structure

Three factors were hypothesized. Exploratory factor analysis resulted in a three-factor solution with a KMO of 0.590 that explained 47 % of the variance (Table 2). We removed items sequentially based on low factor loadings, splitting across multiple factors, or failing to generate a factor analysis solution. This resulted in the extraneous load items being removed entirely. The final model yielded a two-factor solution with a KMO of 0.701 that explained 50 % of the variance. (Table 3) The four items for intrinsic load functioned as a single factor. The single item for germane load formed a second factor. The intrinsic load factor also correlated with the second factor intended to measure germane load (Pearson *r*=0.24, *p* = 0.028).

**Table 2:** Results of exploratory factor analysis of all items

Item	*N*	Mean	SD	Skewness	Kurtosis	Loading factor 1^a^	Loading factor 2^a^	Loading factor 3^a^
Intrinsic load: complexity	86	5.581	1.8261	− 0.168	− 0.343	0.532	− 0.077	0.429
Intrinsic load: acuity	86	7.221	1.7634	− 0.498	0.226	0.552	0.032	0.053
Intrinsic load: amount of clinical information	85	6.31	1.746	− 0.127	− 0.375	0.741	0.184	0.085
Intrinsic load: complexity of clinical decisions	86	6.186	2.3137	− 0.775	0.299	0.750	0.310	− 0.011
Extraneous load: protocol	86	5.372	2.8944	− 0.575	− 0.757	0.042	0.827	− 0.225
Extraneous load: accessibility of information	86	5.093	2.3992	− 0.567	− 0.064	0.104	0.318	0.080
Extraneous load: terminology	86	5.314	2.5449	− 0.401	− 0.285	0.127	0.127	0.733
Extraneous Load: distraction	85	2.52	2.767	1.059	0.127	− 0.308	− 0.176	0.290
Germane Load: understanding	85	4.08	2.803	− 0.035	− 1.034	0.133	0.764	0.083

^a^Extraction method: Principal axis factoring. Rotation method: Varimax with Kaiser normalization.

**Table 3:** Factor loadings for final model

Item	Factor	
	Intrinsic Load	Germane Load
Intrinsic load: complexity	0.50	0.08
Intrinsic load: acuity	0.65	− 0.08
Intrinsic load: amount of clinical information	0.69	0.33
Intrinsic load: complexity of clinical decisions	0.73	0.30
Germane load: understanding	0.09	0.78

Extraction method: principal axis factoring, Rotation method: Varimax with Kaiser normalization.

### Validity evidence related to correlation with other variables

Table 4 summarizes the results of the correlational analyses. It was hypothesized that measures of the load types would correlate with overall load. It was also hypothesized that intrinsic load would vary inversely with performance and experience and that germane load would have a positive association with performance and negative with experience.

**Table 4:** Relationship of cognitive load factors to other variables

Variable	Intrinsic load factor^a^	Germane load factor^b^
Paas single item measure of overall cognitive load	*Pearson correlation coefficient* 0.310 (*N* = 86, *P* = 0.004)	*Pearson correlation coefficient* 0.110 (*N* = 85, *P* = 0.32)
Student self-assessment of handoff’s success	*Pearson correlation coefficient* 0.129 (*N* = 85, *P* = 0.24)	*Pearson correlation coefficient* 0.303 (*N* = 85, *P* = 0.005)
Performance rating by standardized resident	*Pearson correlation coefficient* − 0.140 (*N* = 85, *P* = 0.20)	*Pearson correlation coefficient* 0.107 (*N* = 84, *P* = 0.33)
Low^c^ versus high^d^ handoff experience	*T-test* Low (*N* = 52): M = 26.3 (SD = 5.3) High (*N* = 34): M = 23.6 (SD = 6.3) *P* = 0.036	*T-test* Low (*N* = 52): M = 4.6 (SD = 2.4) High (*N* = 33): M = 3.3 (SD = 3.2) *P* = 0.03

^a^Sum of four contributing factors—range 0–40.

^b^Value of one contributing factor—range 0–10.

^c^Low = prior experience with less than 5 handoffs.

^d^High = prior experience with 5 or more handoffs.

The intrinsic load factor correlated with the Paas single item measure of overall cognitive load (0.31, *p* = 0.004), but did not correlate with the standardized resident’s rating of performance or student self-rating of the handover’s success (*p* = 0.05). The mean for the intrinsic load factor was higher for students with low handover experience (26.3, SD = 5.3) compared with high handover experience (23.6, SD = 6.3, *p* = 0.036) (Table 4).

The germane load factor did not correlate with the Paas single item measure of cognitive load (*p* > 0.05). It did correlate with student self-assessment of handover success (0.303, *p* = 0.005) and was higher in the low experience group (4.6 (SD = 2.4) versus 3.3 (SD = 3.2), *p* = 0.03).

## Discussion

This study represents the first published attempt to measure cognitive load types during a handover. The newly developed instrument, the CLI4H, generated mixed results. While the findings from the exploratory factor analysis are encouraging with respect to intrinsic and germane load, the items for extraneous load performed poorly. The extraneous load items themselves may not be adequate, even though they were tailored to handovers and consistent with the structure of extraneous load items that have performed reasonably well in other settings [13, 18, 24, 28]. This seems to have been the case with respect to the question about how well the student understood the handover protocol. Written comments from the students indicated confusion about this item. Shifting the focus of this item from understanding to ‘clarity about what protocol to use’ may help. In hindsight, ‘clarity’ better captures extraneous load than understanding which relates better to intrinsic load. The item on accessibility of the information used a scale with two concepts—fragmentation and difficulty of organization. This may have led to respondents focusing on different concepts. And the terminology item asks about ‘mental effort to understand’ which may have caused the item to split across extraneous and intrinsic load domains.

In addition to the construction of the extraneous items, the context may have been a primary contributor to the poor performance of these items. The handover occurred in a highly controlled environment in which there were no interruptions or background noise and no fragmentation of information. Consequently, the items focused on distractions and information fragmentation were not tested by the setting. Similarly, the standardized receivers were trained actors who likely did not simulate the ‘give and take’ of an actual clinician-receiver. As a result, we suspect communication was mostly unidirectional, making the item on the clarity of the terminology of questionable applicability. Taken as a whole, these limitations provide guidance for future efforts to measure extraneous load. Response process should be assessed more systematically in the development of new extraneous load items. Items should be tested in environments that better simulate sources of distraction in clinical handovers. Moreover, measurement of certain sources of extraneous load (e.g., clarity of terminology) will require the bi-directional communication of sender and receiver.

The germane load results are promising. However, a single item is not sufficient for confirmatory factor analysis which will be necessary for further validation studies. More items need to be developed and tested. Moreover, germane load may be inadequately specified by our current models. Future items should include metacognition concepts given the similarities between the concept of germane load and metacognition (anticipatory planning, monitoring and adapting action in real time, and reflection and evaluation afterward).

The findings from the correlational analyses provide some additional evidence of validity. Intrinsic load factor showed a positive association with Paas’ measure of cognitive load. While small, the magnitude (0.310) is in a similar range to the correlation found between intrinsic load and Paas’ overall measure (0.347, *p* < 0.01) in a recent study on cognitive load and the use of hypermedia [24]. Still, we expected the correlation to be higher. In addition, the intrinsic load factor was higher for students with less handover experience which is consistent with CLT’s notion that a given task will present less intrinsic load as a learner’s skill increases. Although CLT predicts a negative correlation between intrinsic load and performance, our measure of intrinsic load did not correlate with either of our measures of performance (i.e., self-assessment of success and rating by the standardized resident). This is surprising and inconsistent with other studies [13, 17, 24]. However, the students may not have had sufficient external information and reflection skills to self-assess accurately [42]. In addition, there was very little spread in the performance ratings from the standardized residents (e.g., more than 40 % of the students had the same score of 8). Therefore, the absence of a correlation between intrinsic load and performance likely reflects an inadequate measure of performance—due to the rating tool and/or the raters. The rating tool focused on whether the sender performed each step of the protocol. But variation in performance may arise less from compliance with each step than from the content quality within each step. One group has reported results on the initial testing of a handoff evaluation tool, the Handoff Mini-CEX, which includes a focus on the content quality [43]. Also, the standardized residents who did the performance ratings were actors who typically function as standardized patients and may not have sufficient clinical knowledge to rate the handover. It is less likely but also possible that the learners did not differ enough in their skill or that the intrinsic load of the handover itself was not sufficiently high to generate meaningful differences in performance between different levels of experience.

The study found a negative correlation between the germane load factor and experience. In other words, the less experienced students dedicated more effort to understanding how to perform the handover. Theoretically, performance and learning should improve as germane load increases, again with the proviso that total load does not exceed the learner’s working memory capacity. Some studies have reported a positive correlation [18, 24] while others have not [13, 28]. Our results were similarly mixed—germane load correlated with the subjective measure of success, but not the performance rating by the standardized resident. Given the limitations of self-assessment as a performance measure, the more important point may be the inadequacy of our performance measure (e.g., rating by the standardized residents).

We found only a small association between the intrinsic load factor and the germane load factor, which supports the relative independence of these two constructs—an issue of some controversy in the CLT literature. The triarchic formulation posits that the three load types are separate and thus should not correlate. This perspective places the activities related to schema construction and automation (i.e., learning) in the domain of germane load [12]. Others have argued that intrinsic load encompasses schema acquisition and learning and that germane load represents additional activities that enhance learning such as the conscious application of learning strategies [44]. This perspective defines germane load differently but still maintains germane load as an independent type of load. Still others argue that germane and intrinsic load overlap so significantly that the two categories are redundant and best understood as a single type of load. This latter perspective has gained increasing support from CLT researchers [14, 45]. The results of this study suggest that intrinsic and the single germane load are mostly independent. Yet, other recent studies that have found a third factor have wondered whether the factor may relate to a construct other than germane load [13]. That is a possibility with our results.

Limitations of this study, as addressed above, included an inadequate measure of performance due to non-clinician actors serving as raters and a performance measure that only focused on adherence to a format rather than the quality or accuracy of the information communicated. The simulation also failed to introduce common sources of extraneous load, making it difficult to assess this part of the instrument. These limitations serve as important lessons for subsequent research in this area, especially when the study occurs in a simulated environment such as an OSCE, in which non-clinical actors often rate trainees and occupy important roles, and sources of extraneous load are by design minimized. Future studies should use a meaningful performance measure (such as accuracy or quality of information conveyed). And testing should occur in authentic clinical workplaces or use simulation scenarios that better capture the sources of extraneous load such as interruptions, fragmented information, terminology differences between sender and receiver, and perhaps hierarchies. While reasonable for this initial stage of instrument development to focus on the sender only and the handover of a single patient amongst medical students with experience in handovers, future studies should examine cognitive load in the sender and receiver, sign-out of patient panels, and include trainees with a broader range of experience (e.g., students, residents, and fellows).

## Conclusion

These are the first published results of an instrument designed to measure the cognitive load types associated with a handover. The study employed learners with different levels of experience which allowed the collection of validity evidence beyond factor structure. While preliminary, the results offer some support for the items measuring the intrinsic and germane load constructs. These can be refined and further tested, especially with more germane load items, a better measure of performance, senders and receivers, a broader spectrum of learner levels, and variation in patient complexity. Items for extraneous load require re-building and then testing in an environment that better simulates factors that induce extraneous load. The study’s limitations serve as important insights for future research efforts and represent a set of initial findings upon which future endeavors can build. The ability to measure cognitive load types is critical to our efforts to understand the cognitive load mechanisms of handover procedures. Such a measure will help the field better leverage CLT in order to identify handover procedures that manage intrinsic, extraneous, and germane load in the desired direction, and, thereby, enhance learning, reduce errors and avoid harm to patients.

## References

[1] Horwitz LI, Moin T, Krumholz HM, Wang L, Bradley EH (2008). Consequences of inadequate sign-out for patient care. Arch Intern Med.

[2] Starmer AJ, O’Toole JK, Rosenbluth G (2014). Development, implementation, and dissemination of the I-PASS handoff curriculum: a multisite educational intervention to improve patient handoffs. Acad Med.

[3] Patterson ES, Roth EM, Woods DD, Chow R, Gomes JO (2004). Handoff strategies in settings with high consequences for failure: lessons for health care operations. Int J Qual Health Care.

[4] Wohlauer MV, Arora VM, Horwitz LI (2012). The patient handoff: a comprehensive curricular blueprint for resident education to improve continuity of care. Acad Med.

[5] Starmer AJ, Spector ND, Srivastava R (2014). Changes in medical errors after implementation of a handoff program. N Engl J Med.

[6] Atkinson RC, Shiffrin RM, Kenneth WS, Janet Taylor S (1968). Human memory: a proposed system and its control processes. Psychology of learning and motivation. 2.

[7] Mayer RE (2010). Applying the science of learning to medical education. Med Educ.

[8] Baddeley A (2012). Working memory: theories, models, and controversies. Annu Rev Psychol.

[9] Sweller J (1988). Cognitive load during problem solving: effects on learning. Cogn Sci.

[10] Sweller J, van Merrienboer JJG, Walsh K (2013). Cognitive Load Theory and Instructional Design for Medical Education. The Oxford textbook of medical education.

[11] Cowan N (2001). The magical number 4 in short-term memory: a reconsideration of mental storage capacity. Behav Brain Sci.

[12] Sweller J, van Merrienboer JJG, Paas FGWC (1998). Cognitive architecture and instructional design. Educ Psychol Rev.

[13] Leppink J, Paas F, van Gog T, van der Vleuten CPM, van Merrienboer JJG (2014). Effects of pairs of problems and examples on task performance and different types of cognitive load. Learn Instr.

[14] Sweller J, Ayres PL, Kalyuga S (2011). Cognitive load theory.

[15] van Merriënboer JJG, Sweller J (2005). Cognitive load theory and complex learning: recent developments and future directions. Educ Psychol Rev.

[16] DeLeeuw KE, Mayer RE (2008). A comparison of three measures of cognitive load: evidence for separable measures of intrinsic, extraneous, and germane load. J Educ Psychol.

[17] Ayres P (2006). Using subjective measures to detect variations of intrinsic cognitive load within problems. Learn Instr.

[18] Cierniak G, Scheiter K, Gerjets P (2009). Explaining the split-attention effect: is the reduction of extraneous cognitive load accompanied by an increase in germane cognitive load?. Comput Human Behav.

[19] Eysink TS, de Jong T, Berthold K, Kolloffel B, Opfermann M, Wouters P (2009). Learner performance in multimedia learning arrangements: an analysis across instructional approaches. Am Educ Res J.

[20] Galy E, Cariou M, Mélan C (2012). What is the relationship between mental workload factors and cognitive load types?. Int J Psychophysiol.

[21] Hart SG, Staveland LE, Hancock PA, Meshtaki N (1988). Development of NASA-TLX (Task Load Index): results of empirical and theoretical research. Human mental workload.

[22] Paas FG, Van Merrienboer JJ, Adam JJ (1994). Measurement of cognitive load in instructional research. Percept Mot Skills.

[23] Kluge A, Grauel B, Burkolter D (2013). Combining principles of Cognitive Load Theory and diagnostic error analysis for designing job aids: effects on motivation and diagnostic performance in a process control task. Appl Ergon.

[24] Debue N, van de Leemput C (2014). What does germane load mean? An empirical contribution to the cognitive load theory. Front Psychol.

[25] Paas F, Tuovinen JE, Tabbers H, Van Gerven PWM (2003). Cognitive load measurement as a means to advance cognitive load theory. Educ Psychol.

[26] Haji FA, Rojas D, Childs R, de Ribaupierre S, Dubrowski A (2015). Measuring cognitive load: performance, mental effort and simulation task complexity. Med Educ.

[27] Naismith LM, Cheung JJH, Ringsted C, Cavalcanti RB (2015). Limitations of subjective cognitive load measures in simulation-based procedural training. Med Educ.

[28] Leppink J, Paas F, Van der Vleuten CP, Van Gog T, Van Merrienboer JJ (2013). Development of an instrument for measuring different types of cognitive load. Behav Res Methods.

[29] Hart SG, Staveland LE. Development of NASA-TLX (Task Load Index): results of Empirical and Theoretical Research: Ames Research Center; 1988 [cited 2015 February 14]. https://archive.org/details/nasa_techdoc_20000004342.

[30] Ginns P (2006). Integrating information: a meta-analysis of the spatial contiguity and temporal contiguity effects. Learn Instr.

[31] Kalyuga S, Chandler P, Sweller J (1998). Levels of expertise and instructional design. Hum Factors.

[32] Kester L, Kirschner PA, Van Merriënboer JJG (2005). The management of cognitive load during complex cognitive skill acquisition by means of computer-simulated problem solving. Br J Educ Psychol.

[33] Lafleur A, Côté L, Leppink J (2015). Influences of OSCE design on students’ diagnostic reasoning. Med Educ.

[34] Bergman E, de Bruin A, Vorstenbosch M (2015). Effects of learning content in context on knowledge acquisition and recall: a pretest-posttest control group design. BMC Med Educ.

[35] Downing SM (2003). Validity: on meaningful interpretation of assessment data. Med Educ.

[36] Young JQ, Ten Cate O, O’Sullivan PS, Irby DM. Unpacking the complexity of patient handoffs through the lens of cognitive load theory. Teach Learn Med. (In Press). 10.1080/10401334.2015.1107491 26787089

[37] Salomon G (1984). Television is “easy” and print is “tough”: the differential investment of mental effort in learning as a function of perceptions and attributions. J Educ Psychol.

[38] Artino AR, La Rochelle JS, Dezee KJ, Gehlbach H (2014). Developing questionnaires for educational research: AMEE Guide No. 87. Med Teach.

[39] Paas FG (1992). Training strategies for attaining transfer of problem-solving skill in statistics: a cognitive-load approach. J Educ Psychol.

[40] Chu ES, Reid M, Schulz T, Burden M (2009). A structured handoff program for interns. Acad Med.

[41] Tabachnick BG, Fidell LS (2013). Using multivariate statistics.

[42] Schumacher DJ, Englander R, Carraccio C (2013). Developing the master learner: applying learning theory to the learner, the teacher, and the learning environment. Acad Med.

[43] Arora VM, Berhie S, Horwitz LI, Saathoff M, Staisiunas P, Farnan JM (2014). Using standardized videos to validate a measure of handoff quality: the handoff mini-clinical examination exercise. J Hosp Med.

[44] Schnotz W, Kurschner C (2007). A reconsideration of cognitive load theory. Educ Psychol Rev.

[45] Kalyuga S (2011). Cognitive load theory: how many types of load does it really need?. Educ Psychol Rev.

